# Supplementary figures and images for: Covid-19 in end-stage renal disease patients with renal replacement therapies: A systematic review and meta-analysis
Source: PLoS Negl Trop Dis. 2021 Jun 15;15(6):e0009156. doi: 10.1371/journal.pntd.0009156 (PMC8232454; doi:10.1371/journal.pntd.0009156)

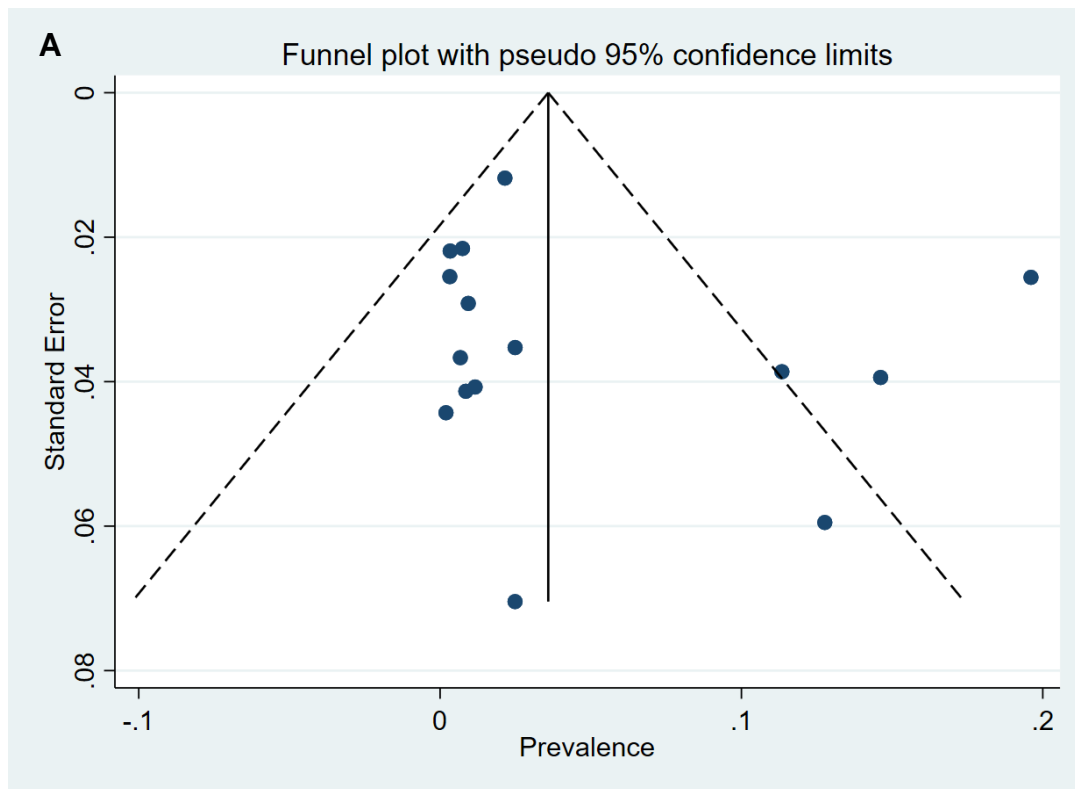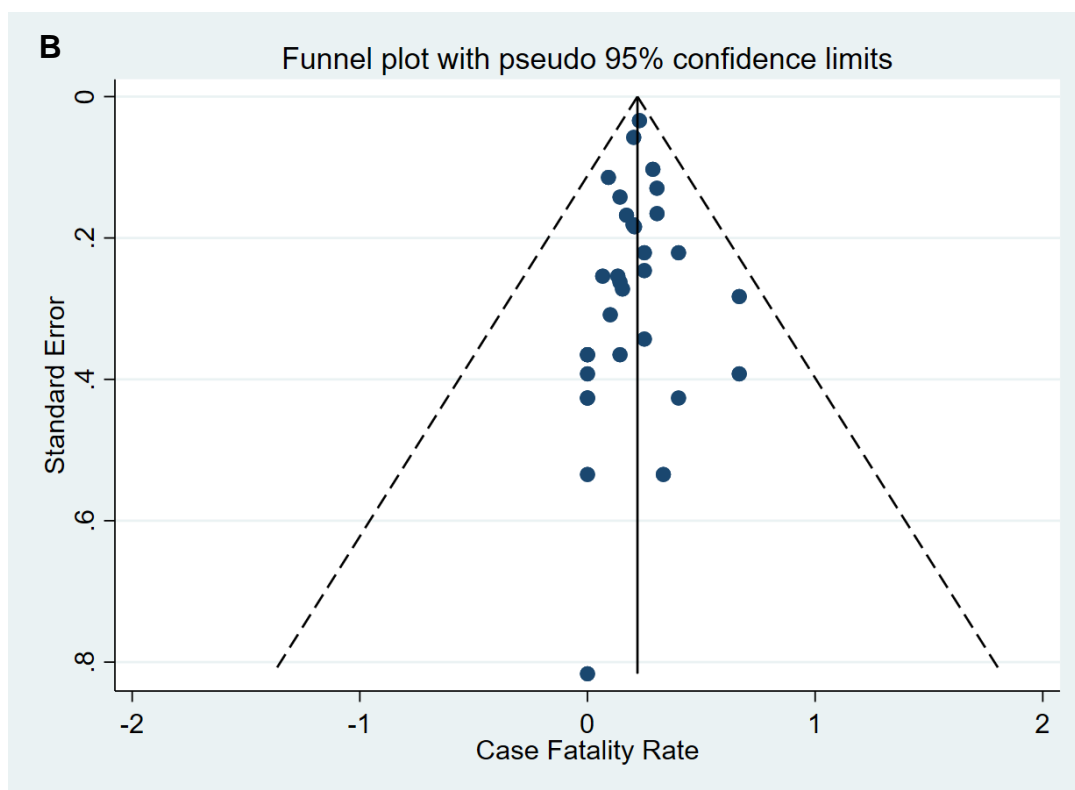

Supplement: S1 Fig — (A) estimated prevalence, (B) estimated case fatality rate. (PDF) [file pntd.0009156.s002.pdf]
