# Supplementary material for: Covid-19 in end-stage renal disease patients with renal replacement therapies: A systematic review and meta-analysis
Source: PLoS Negl Trop Dis. 2021 Jun 15;15(6):e0009156. doi: 10.1371/journal.pntd.0009156 (PMC8232454; doi:10.1371/journal.pntd.0009156)
Supplement: S2 Fig — (A) overall, (B) by country income level, (C) by WHO country region, (D) by RRT modality. (PDF) [file pntd.0009156.s003.pdf]

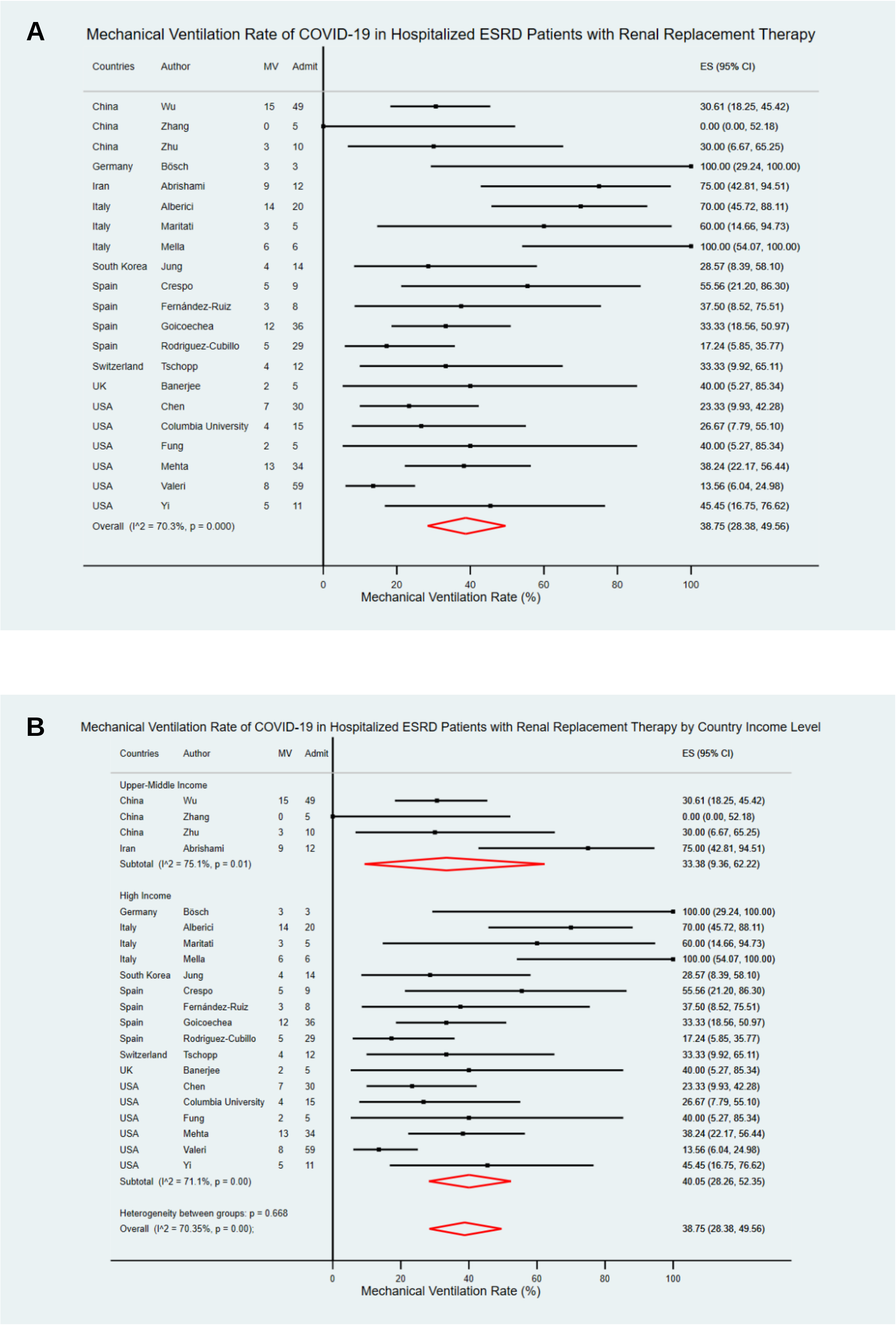
A
Mechanical Ventilation Rate of COVID-19 in Hospitalized ESRD Patients with Renal Replacement Therapy
Countries Author MV Admit ES (95% CI)
China Wu 15 49 30.61 (18.25, 45.42)
China Zhang 0 5 0.00 (0.00, 52.18)
China Zhu 3 10 30.00 (6.67, 65.25)
Germany Bösch 3 3 100.00 (29.24, 100.00)
Iran Abrishami 9 12 75.00 (42.81, 94.51)
Italy Alberici 14 20 70.00 (45.72, 88.11)
Italy Maritati 3 5 60.00 (14.66, 94.73)
Italy Mella 6 6 100.00 (54.07, 100.00)
South Korea Jung 4 14 28.57 (8.39, 58.10)
Spain Crespo 5 9 55.56 (21.20, 86.30)
Spain Fernández-Ruiz 3 8 37.50 (8.52, 75.51)
Spain Goicoechea 12 36 33.33 (18.56, 50.97)
Spain Rodriguez-Cubillo 5 29 17.24 (5.85, 35.77)
Switzerland Tschopp 4 12 33.33 (9.92, 65.11)
UK Banerjee 2 5 40.00 (5.27, 85.34)
USA Chen 7 30 23.33 (9.93, 42.28)
USA Columbia University 4 15 26.67 (7.79, 55.10)
USA Fung 2 5 40.00 (5.27, 85.34)
USA Mehta 13 34 38.24 (22.17, 56.44)
USA Valeri 8 59 13.56 (6.04, 24.98)
USA Yi 5 11 45.45 (16.75, 76.62)
Overall (I^2 = 70.3%, p = 0.000) 38.75 (28.38, 49.56)
0 20 40 60 80 100
Mechanical Ventilation Rate (%)
B
Mechanical Ventilation Rate of COVID-19 in Hospitalized ESRD Patients with Renal Replacement Therapy by Country Income Level
Countries Author MV Admit ES (95% CI)
Upper-Middle Income
China Wu 15 49 30.61 (18.25, 45.42)
China Zhang 0 5 0.00 (0.00, 52.18)
China Zhu 3 10 30.00 (6.67, 65.25)
Iran Abrishami 9 12 75.00 (42.81, 94.51)
Subtotal (I^2 = 75.1%, p = 0.01) 33.38 (9.36, 62.22)
High Income
Germany Bösch 3 3 100.00 (29.24, 100.00)
Italy Alberici 14 20 70.00 (45.72, 88.11)
Italy Maritati 3 5 60.00 (14.66, 94.73)
Italy Mella 6 6 100.00 (54.07, 100.00)
South Korea Jung 4 14 28.57 (8.39, 58.10)
Spain Crespo 5 9 55.56 (21.20, 86.30)
Spain Fernández-Ruiz 3 8 37.50 (8.52, 75.51)
Spain Goicoechea 12 36 33.33 (18.56, 50.97)
Spain Rodriguez-Cubillo 5 29 17.24 (5.85, 35.77)
Switzerland Tschopp 4 12 33.33 (9.92, 65.11)
UK Banerjee 2 5 40.00 (5.27, 85.34)
USA Chen 7 30 23.33 (9.93, 42.28)
USA Columbia University 4 15 26.67 (7.79, 55.10)
USA Fung 2 5 40.00 (5.27, 85.34)
USA Mehta 13 34 38.24 (22.17, 56.44)
USA Valeri 8 59 13.56 (6.04, 24.98)
USA Yi 5 11 45.45 (16.75, 76.62)
Subtotal (I^2 = 71.1%, p = 0.00) 40.05 (28.26, 52.35)
Heterogeneity between groups: p = 0.668
Overall (I^2 = 70.35%, p = 0.00); 38.75 (28.38, 49.56)
0 20 40 60 80 100
Mechanical Ventilation Rate (%)

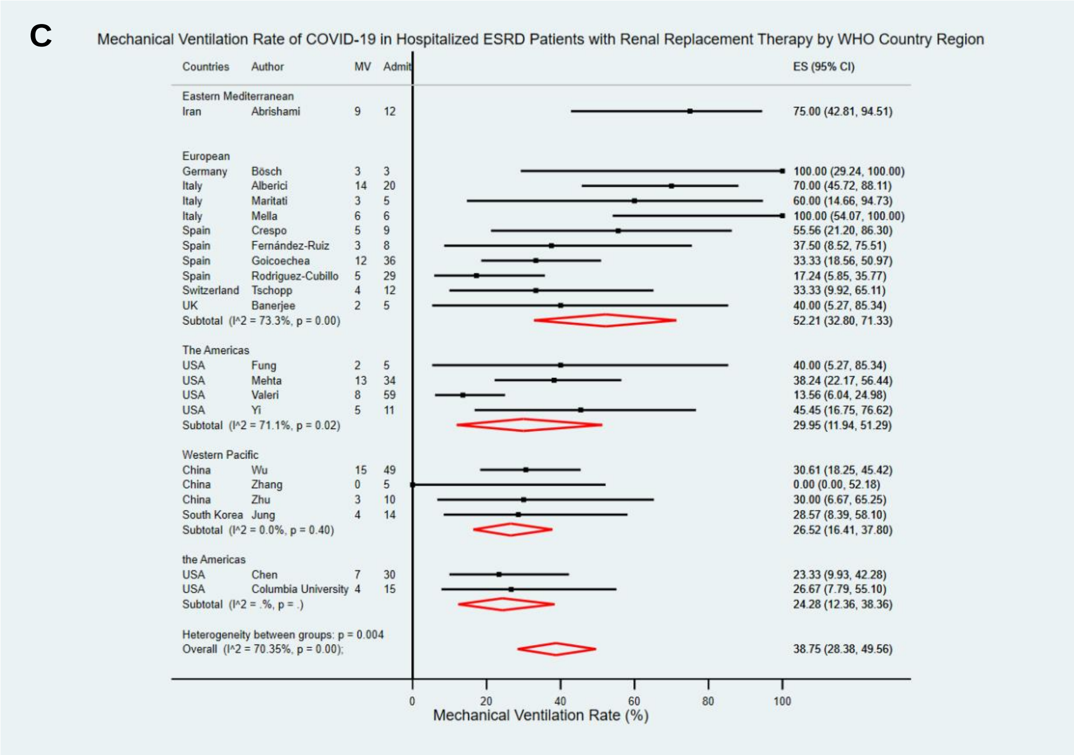
C
Mechanical Ventilation Rate of COVID-19 in Hospitalized ESRD Patients with Renal Replacement Therapy by WHO Country Region
Countries Author MV Admit
ES (95% CI)
Eastern Mediterranean
Iran Abrishami 9 12 75.00 (42.81, 94.51)
European
Germany Bösch 3 3 100.00 (29.24, 100.00)
Italy Alberici 14 20 70.00 (45.72, 88.11)
Italy Maritati 3 5 60.00 (14.66, 94.73)
Italy Mella 6 6 100.00 (54.07, 100.00)
Spain Crespo 5 9 55.56 (21.20, 86.30)
Spain Fernández-Ruiz 3 8 37.50 (8.52, 75.51)
Spain Goicoechea 12 36 33.33 (18.56, 50.97)
Spain Rodriguez-Cubillo 5 29 17.24 (5.85, 35.77)
Switzerland Tschopp 4 12 33.33 (9.92, 65.11)
UK Banerjee 2 5 40.00 (5.27, 85.34)
Subtotal (I^2 = 73.3%, p = 0.00) 52.21 (32.80, 71.33)
The Americas
USA Fung 2 5 40.00 (5.27, 85.34)
USA Mehta 13 34 38.24 (22.17, 56.44)
USA Valeri 8 59 13.56 (6.04, 24.98)
USA Yi 5 11 45.45 (16.75, 76.62)
Subtotal (I^2 = 71.1%, p = 0.02) 29.95 (11.94, 51.29)
Western Pacific
China Wu 15 49 30.61 (18.25, 45.42)
China Zhang 0 5 0.00 (0.00, 52.18)
China Zhu 3 10 30.00 (6.67, 65.25)
South Korea Jung 4 14 28.57 (8.39, 58.10)
Subtotal (I^2 = 0.0%, p = 0.40) 26.52 (16.41, 37.80)
the Americas
USA Chen 7 30 23.33 (9.93, 42.28)
USA Columbia University 4 15 26.67 (7.79, 55.10)
Subtotal (I^2 = .%, p = .) 24.28 (12.36, 38.36)
Heterogeneity between groups: p = 0.004
Overall (I^2 = 70.35%, p = 0.00); 38.75 (28.38, 49.56)
0 20 40 60 80 100
Mechanical Ventilation Rate (%)

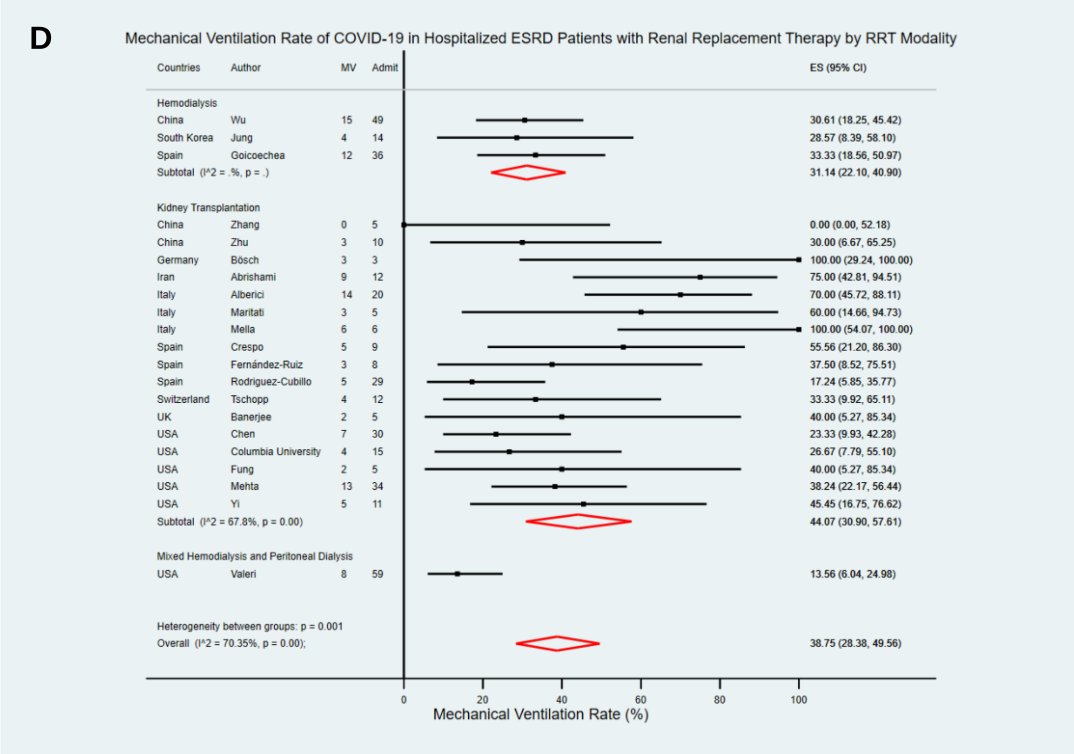
D
Mechanical Ventilation Rate of COVID-19 in Hospitalized ESRD Patients with Renal Replacement Therapy by RRT Modality
Countries Author MV Admit
ES (95% CI)
Hemodialysis
China Wu 15 49 30.61 (18.25, 45.42)
South Korea Jung 4 14 28.57 (8.39, 58.10)
Spain Goicoechea 12 36 33.33 (18.56, 50.97)
Subtotal (I^2 = .%, p = .) 31.14 (22.10, 40.90)
Kidney Transplantation
China Zhang 0 5 0.00 (0.00, 52.18)
China Zhu 3 10 30.00 (6.67, 65.25)
Germany Bösch 3 3 100.00 (29.24, 100.00)
Iran Abrishami 9 12 75.00 (42.81, 94.51)
Italy Alberici 14 20 70.00 (45.72, 88.11)
Italy Maritati 3 5 60.00 (14.66, 94.73)
Italy Mella 6 6 100.00 (54.07, 100.00)
Spain Crespo 5 9 55.56 (21.20, 86.30)
Spain Fernández-Ruiz 3 8 37.50 (8.52, 75.51)
Spain Rodriguez-Cubillo 5 29 17.24 (5.85, 35.77)
Switzerland Tschopp 4 12 33.33 (9.92, 65.11)
UK Banerjee 2 5 40.00 (5.27, 85.34)
USA Chen 7 30 23.33 (9.93, 42.28)
USA Columbia University 4 15 26.67 (7.79, 55.10)
USA Fung 2 5 40.00 (5.27, 85.34)
USA Mehta 13 34 38.24 (22.17, 56.44)
USA Yi 5 11 45.45 (16.75, 76.62)
Subtotal (I^2 = 67.8%, p = 0.00) 44.07 (30.90, 57.61)
Mixed Hemodialysis and Peritoneal Dialysis
USA Valeri 8 59 13.56 (6.04, 24.98)
Heterogeneity between groups: p = 0.001
Overall (I^2 = 70.35%, p = 0.00); 38.75 (28.38, 49.56)
0 20 40 60 80 100
Mechanical Ventilation Rate (%)
