# Supplementary material for: Covid-19 in end-stage renal disease patients with renal replacement therapies: A systematic review and meta-analysis
Source: PLoS Negl Trop Dis. 2021 Jun 15;15(6):e0009156. doi: 10.1371/journal.pntd.0009156 (PMC8232454; doi:10.1371/journal.pntd.0009156)
Supplement: S3 Fig — (A) overall, (B) by country income level, (C) by WHO country region, (D) by RRT modality. (PDF) [file pntd.0009156.s004.pdf]

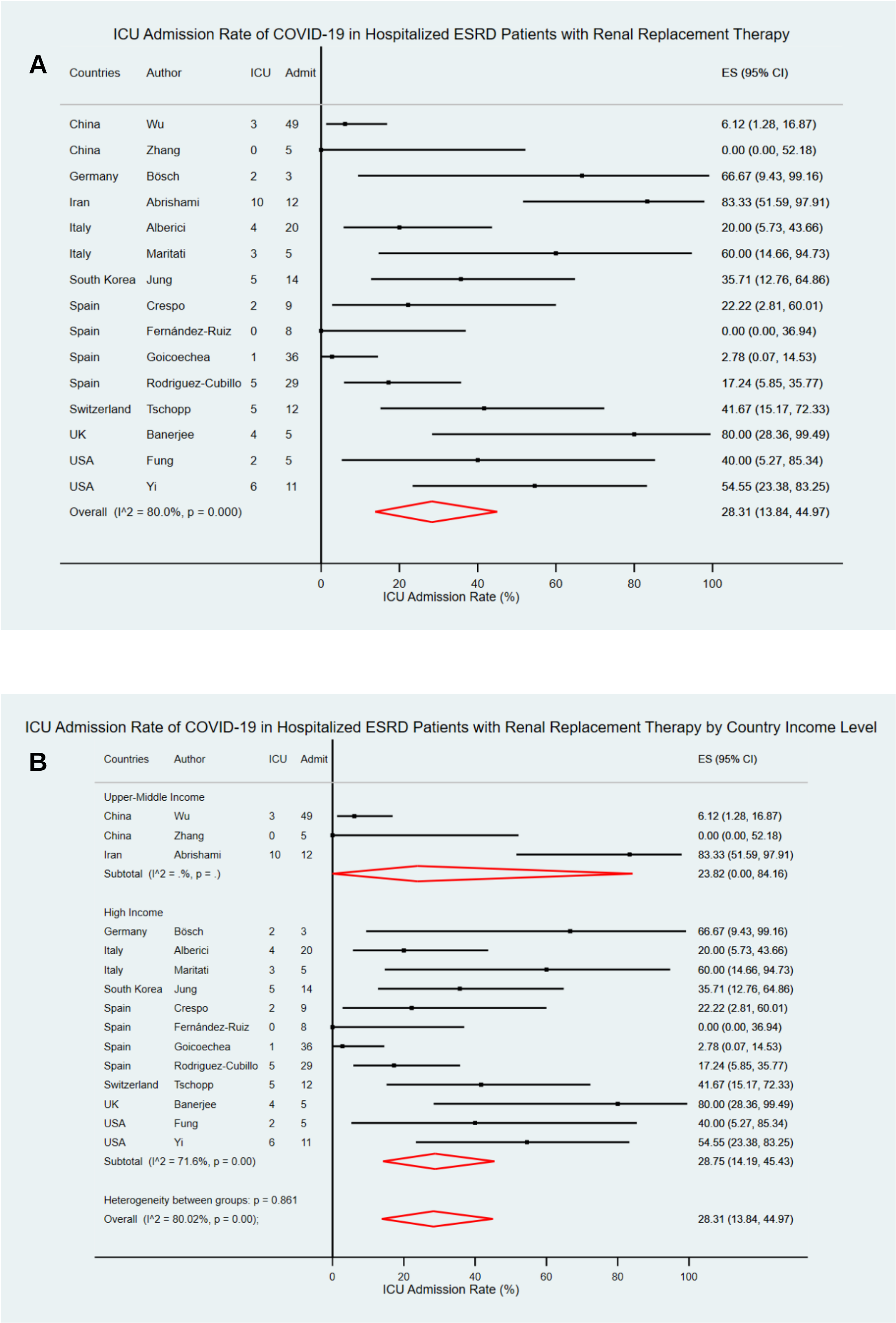
A
ICU Admission Rate of COVID-19 in Hospitalized ESRD Patients with Renal Replacement Therapy
Countries Author ICU Admit ES (95% CI)
China Wu 3 49 6.12 (1.28, 16.87)
China Zhang 0 5 0.00 (0.00, 52.18)
Germany Bösch 2 3 66.67 (9.43, 99.16)
Iran Abrishami 10 12 83.33 (51.59, 97.91)
Italy Alberici 4 20 20.00 (5.73, 43.66)
Italy Maritati 3 5 60.00 (14.66, 94.73)
South Korea Jung 5 14 35.71 (12.76, 64.86)
Spain Crespo 2 9 22.22 (2.81, 60.01)
Spain Fernández-Ruiz 0 8 0.00 (0.00, 36.94)
Spain Goicoechea 1 36 2.78 (0.07, 14.53)
Spain Rodriguez-Cubillo 5 29 17.24 (5.85, 35.77)
Switzerland Tschopp 5 12 41.67 (15.17, 72.33)
UK Banerjee 4 5 80.00 (28.36, 99.49)
USA Fung 2 5 40.00 (5.27, 85.34)
USA Yi 6 11 54.55 (23.38, 83.25)
Overall (I^2 = 80.0%, p = 0.000) 28.31 (13.84, 44.97)
0 20 40 60 80 100
ICU Admission Rate (%)
B
ICU Admission Rate of COVID-19 in Hospitalized ESRD Patients with Renal Replacement Therapy by Country Income Level
Countries Author ICU Admit ES (95% CI)
Upper-Middle Income
China Wu 3 49 6.12 (1.28, 16.87)
China Zhang 0 5 0.00 (0.00, 52.18)
Iran Abrishami 10 12 83.33 (51.59, 97.91)
Subtotal (I^2 = .%, p = .) 23.82 (0.00, 84.16)
High Income
Germany Bösch 2 3 66.67 (9.43, 99.16)
Italy Alberici 4 20 20.00 (5.73, 43.66)
Italy Maritati 3 5 60.00 (14.66, 94.73)
South Korea Jung 5 14 35.71 (12.76, 64.86)
Spain Crespo 2 9 22.22 (2.81, 60.01)
Spain Fernández-Ruiz 0 8 0.00 (0.00, 36.94)
Spain Goicoechea 1 36 2.78 (0.07, 14.53)
Spain Rodriguez-Cubillo 5 29 17.24 (5.85, 35.77)
Switzerland Tschopp 5 12 41.67 (15.17, 72.33)
UK Banerjee 4 5 80.00 (28.36, 99.49)
USA Fung 2 5 40.00 (5.27, 85.34)
USA Yi 6 11 54.55 (23.38, 83.25)
Subtotal (I^2 = 71.6%, p = 0.00) 28.75 (14.19, 45.43)
Heterogeneity between groups: p = 0.861
Overall (I^2 = 80.02%, p = 0.00); 28.31 (13.84, 44.97)
0 20 40 60 80 100
ICU Admission Rate (%)

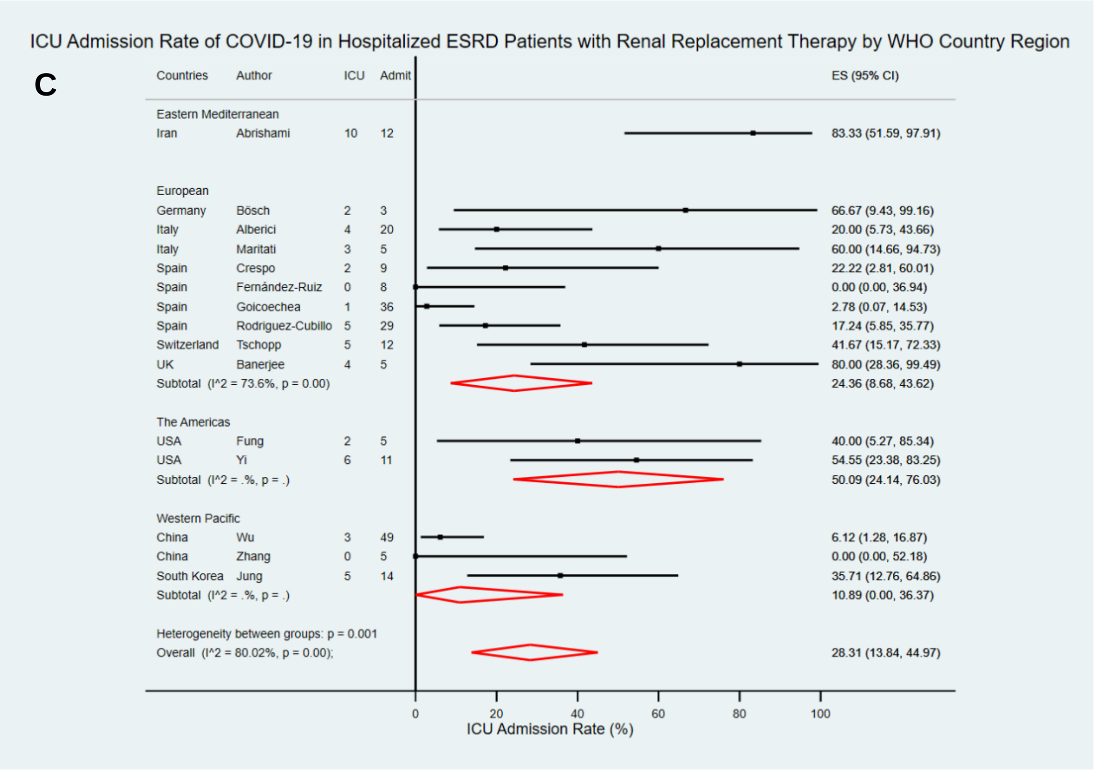
ICU Admission Rate of COVID-19 in Hospitalized ESRD Patients with Renal Replacement Therapy by WHO Country Region
C
Countries Author ICU Admit ES (95% CI)
Eastern Mediterranean
Iran Abrishami 10 12 83.33 (51.59, 97.91)
European
Germany Bösch 2 3 66.67 (9.43, 99.16)
Italy Alberici 4 20 20.00 (5.73, 43.66)
Italy Maritati 3 5 60.00 (14.66, 94.73)
Spain Crespo 2 9 22.22 (2.81, 60.01)
Spain Fernández-Ruiz 0 8 0.00 (0.00, 36.94)
Spain Goicoechea 1 36 2.78 (0.07, 14.53)
Spain Rodriguez-Cubillo 5 29 17.24 (5.85, 35.77)
Switzerland Tschopp 5 12 41.67 (15.17, 72.33)
UK Banerjee 4 5 80.00 (28.36, 99.49)
Subtotal (I^2 = 73.6%, p = 0.00) 24.36 (8.68, 43.62)
The Americas
USA Fung 2 5 40.00 (5.27, 85.34)
USA Yi 6 11 54.55 (23.38, 83.25)
Subtotal (I^2 = .%, p = .) 50.09 (24.14, 76.03)
Western Pacific
China Wu 3 49 6.12 (1.28, 16.87)
China Zhang 0 5 0.00 (0.00, 52.18)
South Korea Jung 5 14 35.71 (12.76, 64.86)
Subtotal (I^2 = .%, p = .) 10.89 (0.00, 36.37)
Heterogeneity between groups: p = 0.001
Overall (I^2 = 80.02%, p = 0.00); 28.31 (13.84, 44.97)
0 20 40 60 80 100
ICU Admission Rate (%)

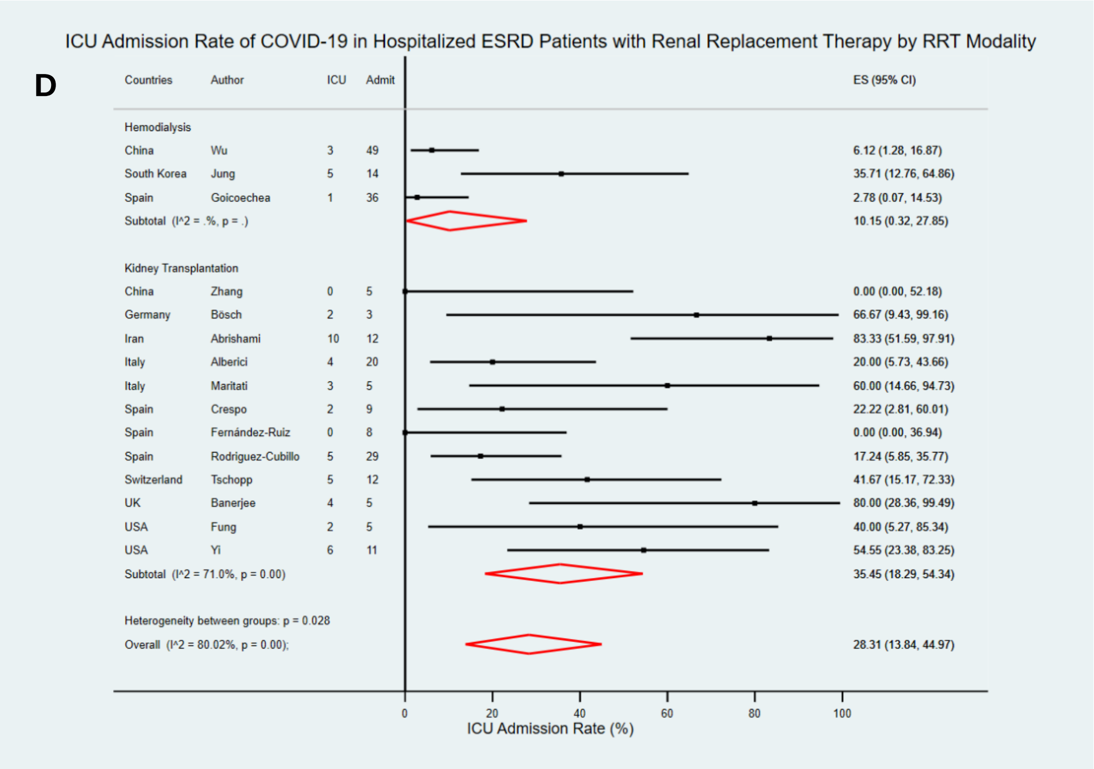
ICU Admission Rate of COVID-19 in Hospitalized ESRD Patients with Renal Replacement Therapy by RRT Modality
D
Countries Author ICU Admit ES (95% CI)
Hemodialysis
China Wu 3 49 6.12 (1.28, 16.87)
South Korea Jung 5 14 35.71 (12.76, 64.86)
Spain Goicoechea 1 36 2.78 (0.07, 14.53)
Subtotal (I^2 = .%, p = .) 10.15 (0.32, 27.85)
Kidney Transplantation
China Zhang 0 5 0.00 (0.00, 52.18)
Germany Bösch 2 3 66.67 (9.43, 99.16)
Iran Abrishami 10 12 83.33 (51.59, 97.91)
Italy Alberici 4 20 20.00 (5.73, 43.66)
Italy Maritati 3 5 60.00 (14.66, 94.73)
Spain Crespo 2 9 22.22 (2.81, 60.01)
Spain Fernández-Ruiz 0 8 0.00 (0.00, 36.94)
Spain Rodriguez-Cubillo 5 29 17.24 (5.85, 35.77)
Switzerland Tschopp 5 12 41.67 (15.17, 72.33)
UK Banerjee 4 5 80.00 (28.36, 99.49)
USA Fung 2 5 40.00 (5.27, 85.34)
USA Yi 6 11 54.55 (23.38, 83.25)
Subtotal (I^2 = 71.0%, p = 0.00) 35.45 (18.29, 54.34)
Heterogeneity between groups: p = 0.028
Overall (I^2 = 80.02%, p = 0.00); 28.31 (13.84, 44.97)
0 20 40 60 80 100
ICU Admission Rate (%)
