# Supplementary material for: Covid-19 in end-stage renal disease patients with renal replacement therapies: A systematic review and meta-analysis
Source: PLoS Negl Trop Dis. 2021 Jun 15;15(6):e0009156. doi: 10.1371/journal.pntd.0009156 (PMC8232454; doi:10.1371/journal.pntd.0009156)
Supplement: Attachment — Submitted filename: Response to Reviewer COVIDRRT 5.2 PLOS NTD KP clean.docx [file pntd.0009156.s007.docx]

Dear Editor,

We thank you and the reviewers for the comments and suggestions. Please find our point-by-point responses below:

Editor: Thank you very much for submitting your manuscript "Covid-19 in end-stage renal disease patients with renal replacement therapies: a systematic review and meta-analysis" for consideration at PLOS Neglected Tropical Diseases. As with all papers reviewed by the journal, your manuscript was reviewed by members of the editorial board and by several independent reviewers. The reviewers appreciated the attention to an important topic. Based on the reviews, we are likely to accept this manuscript for publication, providing that you modify the manuscript according to the review recommendations.

Response: Thank you for your time and consideration. The manuscript was revised according to the review recommendations to meet PLOS Neglected Tropical Diseases’ standard.

Reviewer #1: Nopsopon and colleagues performed a systematic review and meta-analysis on mortality in patients with end-stage kidney disease and maintenance dialysis. This is a timely and important work.

Response: Thank you very much for your compliment.

Reviewer #1: Why were patients with kidney transplantation considered as ESRD patients? I would assume that they are a separate group. The mechanisms of COVID-19 disease severity are likely to be different from uremia in patients with kidney transplantation. Unless the authors don't have a valid reason, I would recommend to exclude the group

Response: Thank you for pointing out the issue. This meta-analysis aimed to evaluate the prevalence and case fatality rate of COVID-19 in ESRD patients who received different RRT modalities. While the mechanisms of COVID-19 severity might differ between patients with kidney transplantation and patients with dialysis, all patients with kidney transplantation were ESRD patients before transplantation, and kidney transplantation was one of RRT modality. Thus, patients with kidney transplantation should be considered as ESRD patient who received RRT (ESRD with RRT). Excluding patients with kidney transplantation might shed less light on actual situation for COVID-19 infection in ESRD with RRT patients.

We do agree that the different mechanisms of COVID-19 infection and severity between transplant and dialysis patients could affect the outcomes. The subgroup analysis across RRT modalities (separate Peritoneal dialysis, Hemodialysis, Kidney transplantation, and Mixed RRT) on Fig.5 and Fig.9 with elaborated explanation was included in the Result section. Also, we added a discussion on mechanisms of COVID-19 infection and severity in the Discussion section.

Reviewer #1: Why did the authors only include studies up to June 2020 (i.e. 1 year before the submission of this work)?

Response: Because we performed the systematic search on June 30, 2020 and spent a few months on preparing and submitting the manuscript.

Reviewer #1: The manuscript requires serious proofreading to improve the readability

Response: Thank you for your comment. The manuscript was revised to improve the readability as suggested.

Reviewer #1: Figure legends are normally placed at the end of the manuscript

Response: Thank you for your comment. Figure legends were moved to the end of the manuscript.

Reviewer #2: METHOD: “We regarded level of heterogeneity for I2 statistic as defined in chapter 9 of the Cochrane Handbook for Systematic Reviews of Interventions: 0–40% might not be important; 30–60% may represent moderate heterogeneity; 50–90% may represent substantial heterogeneity; 75–100% considerable heterogeneity. There was a problem in the criteria.

Response: Thank you for pointing out the issue. The criteria were described in Cochrane Handbook for Systematic Reviews of Interventions as presented in the manuscript. There was actual overlapping in range between categories as the criteria were only a rough guide to interpretation. While thresholds for the interpretation of I^2^ can be misleading, we also considered magnitude and direction of effects, and strength of evidence for heterogeneity. An additional explanation was added in the Method section.

Reviewer #2: ABSTRACT and DISCUSSION: “There was no systematic study on the infection and mortality of the SARS-CoV-2 infection in ESRD patients who are on RRT.” A recent meta-analysis and systematic review found that the incidence of coronavirus infection was 7.7% (95% CI: 4.9%-11.1%) in prevalent hemodialysis patients with an overall mortality rate of 26.2% (95% CI: 20.6%-32.6%) (Ren Fail. 2020 Nov 9;43(1):1-15). The difference between this article and the review by Dr. Zhou et al. should be discussed in the discussion part.

Response: Thank you for your suggestion. Dr.Zhou et al. article was discussed in the Discussion section regarding COVID-19 infection and mortality in pre-existing hemodialysis patients. The concerned sentence in the Abstract was revised.

Reviewer #2: 2 line 352 ‘KTwere’ , line 363 ‘CFRacross’.

Response: Thank you for your comment. Typos were revised throughout the manuscript.

Reviewer #2: 3 Subgroup analysis could add one according to the tropical and frigid zone if possible.

Response: Thank you for your suggestion. Unfortunately, there was no included study conducted in the frigid zone. Of six USA studies, four studies were conducted in New York (Chen, Columbia U, Mehta, and Valeri), one in San Francisco (Fung) and one in Texas (Yi) where those states were not in frigid zone of USA.

Reviewer #3: This is an interesting, well designed and well conducted meta-analysis about risk of COVID in ESRD/RRT. Authors conclude in line with a majority of studies that patients under RRT are in higher risk for severe COVID and mortality rates as high as 18%. I consider it as a very valuable paper and probably will be highly referenced since it resume current evidence in risk of mortality for ESRD.

Response: Thank you for your compliment.

Reviewer #3: Corrections that should be done and suggestions: Since the aim of study is to include different regions of the world, I suggest to include an EU reference with more that 1600 COVID cases, similar to REF 5 and to support comments about AKI-COVID in page 4 line 87 REF 12-14 (Nephrology Dialysis Transplantation, Volume 35, Issue 8, August 2020, Pages 1353–1361, https://doi.org/10.1093/ndt/gfaa189)

Response: Thank you for your suggestion. The EU reference (Nephrology Dialysis Transplantation, Volume 35, Issue 8, August 2020, Pages 1353–1361, https://doi.org/10.1093/ndt/gfaa189) was included in Introduction section as suggested.

Reviewer #3: Methods (details concerning author consultation) seems to be duplicate in page 5 line 121 and page 6 line 137. Correct it please.

Response: Thank you for pointing out the issue. The duplicate was removed from the Quality assessment subheading.

Reviewer #3: It would be useful to give information about non-responding authors rates.

Response: Thank you for your comment. While we mentioned about author consultation in case of incompletely reported data in both protocol and manuscript. Fortunately, we did not have to consult the authors because all included studies provided sufficient important data for primary outcome analysis.

Reviewer #3: Page 11 line 213 typo error, space after brackets

Response: Thank you for your comment. The typos were revised throughout the manuscript.

Reviewer #3: Some of the selected papers include data regarding the policy of exclusion of ESRD patient for Critical Unit. This point is highly relevant and should be added and commented (i.e. after data in page 6 line 332).

Response: Thank you for pointing out the issue. While we concur that the policy of exclusion of ESRD patients is important; however, the studies with hospital admission information did not have exclusion criteria of ESRD patients for critical unit/intensive care unit whereas only 57.7% (15 of 26) provided sufficient information about intensive care unit stay. Additional data was provided in the Result section, and additional discussion was provided in the Discussion section as limitation.

Reviewer #3: A majority of studies came from the 1st pandemic wave. Some comment could be done regarding potential differences with subsequent waves and the external validity of these results.

Response: Thank you for pointing out the issue. An additional discussion was provided in the Discussion section as limitation.

We hope that our responses are satisfactory. Should there be anything that might improve our work, please kindly inform us. Thank you very much for your kind consideration.

Best Regards,

Assoc. Prof. Dr. Krit Pongpirul, MD, MPH, PhD.

On behalf of the authors
